# Cortical Spiking Network Interfaced with Virtual Musculoskeletal Arm and Robotic Arm

**DOI:** 10.3389/fnbot.2015.00013

**Published:** 2015-11-25

**Authors:** Salvador Dura-Bernal, Xianlian Zhou, Samuel A. Neymotin, Andrzej Przekwas, Joseph T. Francis, William W. Lytton

**Affiliations:** ^1^Department of Physiology and Pharmacology, State University of New York Downstate Medical CenterBrooklyn, NY, USA; ^2^CFD Research CorporationHuntsville, AL, USA; ^3^The Robert Furchgott Center for Neural and Behavioral Science, State University of New York Downstate Medical CenterBrooklyn, NY, USA; ^4^Joint Graduate Program in Biomedical Engineering, State University of New York Downstate and Polytechnic Institute of New York UniversityBrooklyn, NY, USA; ^5^Department of Neurology, State University of New York Downstate Medical CenterBrooklyn, NY, USA; ^6^Department of Neurology, Kings County Hospital CenterBrooklyn, NY, USA

**Keywords:** spiking network, biomimetic, musculoskeletal arm, virtual arm, robot arm, reaching, sensorimotor, neuroprosthetics

## Abstract

Embedding computational models in the physical world is a critical step towards constraining their behavior and building practical applications. Here we aim to drive a realistic musculoskeletal arm model using a biomimetic cortical spiking model, and make a robot arm reproduce the same trajectories in real time. Our cortical model consisted of a 3-layered cortex, composed of several hundred spiking model-neurons, which display physiologically realistic dynamics. We interconnected the cortical model to a two-joint musculoskeletal model of a human arm, with realistic anatomical and biomechanical properties. The virtual arm received muscle excitations from the neuronal model, and fed back proprioceptive information, forming a closed-loop system. The cortical model was trained using spike timing-dependent reinforcement learning to drive the virtual arm in a 2D reaching task. Limb position was used to simultaneously control a robot arm using an improved network interface. Virtual arm muscle activations responded to motoneuron firing rates, with virtual arm muscles lengths encoded via population coding in the proprioceptive population. After training, the virtual arm performed reaching movements which were smoother and more realistic than those obtained using a simplistic arm model. This system provided access to both spiking network properties and to arm biophysical properties, including muscle forces. The use of a musculoskeletal virtual arm and the improved control system allowed the robot arm to perform movements which were smoother than those reported in our previous paper using a simplistic arm. This work provides a novel approach consisting of bidirectionally connecting a cortical model to a realistic virtual arm, and using the system output to drive a robotic arm in real time. Our techniques are applicable to the future development of brain neuroprosthetic control systems, and may enable enhanced brain-machine interfaces with the possibility for finer control of limb prosthetics.

## 1. Introduction

Embodiment of the nervous system in the physical world confers both constraints and advantages on learning and behavior (Almássy et al., 1998). For example, exposure to the environment allows selection of particular neuronal and physical dynamics that are best suited to produce desired behaviors through the agency of a limb or other effector (Edelman, 1987). Cortical computational models can similarly benefit from such embodiment and allow us to study the interactions between neural activity and behavior.

In previous work we developed biomimetic cortical models that replicate basic brain processes of spiking dynamics and sensorimotor learning (Neymotin et al., 2011). We demonstrated the use of a spike-timing-dependent reinforcement learning training method in allowing a sensorimotor cortical model to learn to control a one- (Chadderdon et al., 2012) and two- (Neymotin et al., 2013) degree-of-freedom (DOF) simple arm to reach a target. Recently, we used the output of the two-DOF model to drive a robotic arm in real-time via a network interface (Dura-Bernal et al., 2014a). Development of this interface demonstrated the difficulty of matching the alien dynamics of a mechanical arm directly to a biomimetic system, as well as the limitations of the highly-simplified kinematic arm that was employed.

A number of questions emerged from these previous studies which motivated the work presented in this paper. How would the system behave with a more realistic arm model? Would it improve the reaching trajectories and the corresponding robot arm movements? How would the spiking network interact with, and learn to control, the more complex arm model? To address these questions we replaced the previous simple kinematic arm with a detailed model of a musculoskeletal arm, and adapted the interface and learning mechanisms to the new arm.

The new virtual arm includes rigid bodies (bones), joints, muscles and tendons. Its kinematics are governed by a set of ordinary differential equations (ODEs) that compute muscle activation, length, and force, as well as arm motions and forces, at millisecond resolution. The cortical model was interfaced with the virtual arm by exciting the arm muscles using the spiking output from the motor neuron population. Proprioceptive information from muscle lengths then provided activation for a proprioceptive neural population. Arm joint angles were also fed back to the biomimetic model and used to calculate the error signal during the reinforcement learning-based training phase. Results showed that, after training, the reaching trajectories of the virtual musculoskeletal arm were smoother than those of the previous simple arm. Additionally, we developed an improved real-time control interface to drive the robotic arm using the output position information of the musculoskeletal arm. This improved control interface, together with the new virtual arm, lead to smoother and more realistic robot arm movements as compared to our previous implementation.

The main contribution of this work is to extend a system where a biomimetic spiking network learns to control a virtual arm and a robotic arm, by replacing the existing simple virtual arm with a more realistic musculoskeletal model. The relevance of the study is two-fold. First, we demonstrate that increasing the realism of the arm model reduces the arm trajectory jerk and results in velocity profiles closer to biology. These effects are also reflected in the smoother robot movements. This is an important step towards building brain-machine interfaces that can employ biomimetic network models to interact with real brain signals and provide real-time prosthetic arm control (Sanchez et al., 2012). Second, the new virtual arm imposes more accurate physical constraints on the cortical model, allowing us to explore the system under more realistic conditions. The interactions we model here take place across several spatial and temporal scales that involve both the brain and the arm: cell (e.g., spike generation and synaptic adaptation), network (e.g., excitatory-inhibitory balance, oscillations), and behavior (e.g., muscle excitation, torques, elasticity). The ability to directly access and manipulate all the elements of the system provides a useful tool for neuroscience and neural engineering.

## 2. Materials and methods

**Figure 1:**
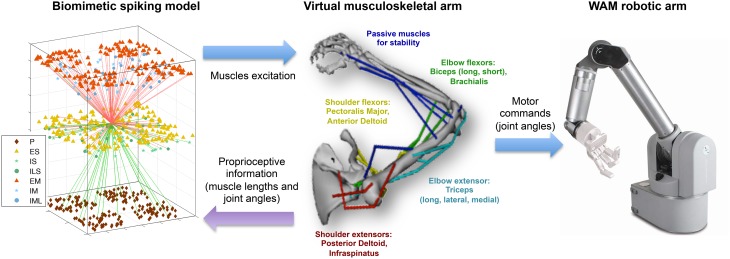
**Overview of the multiscale biomimetic system interfacing the spiking neuronal model with the virtual musculoskeletal arm and the Whole Arm Manipulator (WAM) robot**. The virtual arm receives neural excitation from the biomimetic model and feeds back the joint angles, used in the reinforcement learning algorithm, and the muscle lengths, used as part of the sensorimotor mapping. The joint angles are also used to drive the WAM robotic arm in real time. For the neuronal model, all incoming (green) and outgoing (red) connections of a single ES neuron are shown, and the different cell types are shown in the legend. For the virtual arm, muscles are labeled in the corresponding color.

Our overall system is closed loop with an open loop mirroring from the virtual arm to the physical robotic arm (Figure 1). We first describe here the closed loop components: the spiking neuronal model and the virtual musculoskeletal arm. We then go on to detail the interface between this closed-loop learning system and the robotic arm.

### 2.1. Virtual musculoskeletal arm

The starting point for our virtual arm was the biomechanical model of the upper extremity musculoskeletal system developed by Holzbaur et al. (2005), downloadable from the SimTK website (http://simtk.org/home/up-ext-model), but adapted for our purposes by CFD Research Corporation (http://www.cfdrc.com). The kinematics of each joint and the force-generating parameters for each muscle in this system have been derived from anatomical and physiological studies, and represent those of a human adult male of average size. The moment arms generated by the model captured the primary features of the upper extremity geometry and mechanics, including complex joint coupling effects, where the mechanics of a given joint depends on the posture of adjacent joints.

This model was modified to include only two degrees of freedom (DOFs): shoulder and elbow joint rotation in the horizontal plane. The resulting model includes the following skeletal rigid bodies, where muscles are anchored: ground, thorax, clavicle, scapula, humerus, ulna, radius, and hand. Major active muscles responsible for shoulder and elbow motion were retained, including shoulder extensor muscles (posterior deltoid, infraspinatus, lattisimus dorsi, and teres minor); shoulder flexor muscles (anterior deltoid, pectoralis major, and corachobrachialis); elbow extensor muscles (triceps); elbow flexor muscles (biceps and brachialis). Several additional muscles (lateral deltoid, anconeous, brachioradialis, extensor carpi radialis longus, and pronator teres) were set passively for joint stability. Muscles with multiple heads (deltoid, pectoralis, lattisimus dorsi, triceps, biceps) had the muscle branches appropriately connected to the different insertion and origin points of the skeleton, but were grouped and controlled by the same input signal for simplicity. The final revised 2-DOF arm model is stored in a standard XML-based file included with the full model on modelDB: http://modeldb.yale.edu/183014.

Virtual arm dynamics were implemented using an extension of the Hill-type muscle model (Zajac, 1989; Schutte et al., 1993; Thelen et al., 2003), which required four muscle parameters derived from anatomical studies (Holzbaur et al., 2005): optimal fiber length, peak force, tendon slack length, and pennation angle (angling of individual fibers at the tendon insertion).

Each Hill-type muscle model can be can be described by a lumped-parameter model that accounts for the force-length-velocity properties of muscle (fiber) and the elastic properties of tendon (serially linked with the muscle). Overall muscle-tendon force depended on current muscle length (*l*_*m*_), overall muscle-tendon length (*l*_*mt*_), muscle fiber activation (*a*), and contraction velocity (*v*), as well as the balance between muscle and tendon. At every time step (typically set to 1ms), given the input neural excitation (μ) to each muscle (a value between 0: minimum and 1: maximum), the model calculated the current muscle activation, according to an ODE governed by the muscle excitation-activation mechanism

$$\dot{a}=\{\begin{array}{ll} \left(\mu \tau_{1}+\tau_{2})/\tau \cdot (\mu -a\right) & \mu \geq \text{a}\\ \tau_{2}/\tau \cdot (\mu -a) & \mu <\text{a} \end{array}$$

where τ_1_ and τ_2_ are time constants of ramping up and down activation, respectively, τ is a scale factor for normalizing time with a default value of 100 ms. Once the activation is determined, the muscle (fiber) contraction velocity (${\dot{l}}_{m}$), i.e., the rate of change of muscle length, can be determined from the force balance between muscle and tendon, which dictates the muscle force produced from the muscle force-velocity relation (*f*_*v*_) and consequently results in ${\dot{l}}_{m}=f_{v}^{-1}\left(l_{m},l_{mt},a\right)$. During time-advancing integration, both activation and contraction velocity are integrated numerically and then used to computer the overall muscle-tendon force acting on bones.

The acceleration, position, and velocity of each of the joints are then computed with a recursive Newton-Euler algorithm of robot dynamics (Featherstone and Orin, 2000), taking into account of all driving forces from muscles. The model was implemented in C++, and the open source OpenSceneGraph rendering engine (http://www.openscenegraph.org/) was used for 3D visualization.

For comparison purposes we also evaluated the system using our previous much simpler kinematic model (Neymotin et al., 2013), which was characterized using two line segments (upper arm and forearm), joint angles for shoulder and elbow, and extensor and flexor excitation values for each joint. Joint angle change was calculated as the difference between extensor and flexor excitations (normalized spike counts), and muscle lengths were calculated as a simple linear transformation of the joint angles. No realistic anatomical, biomechanical or kinetic arm features were involved.

### 2.2. Biomimetic spiking neuronal model

Individual neurons were modeled as event-driven, rule-based units for speed of simulation. Given finite computing resources, a tradeoff must be made between the complexity of neurons vs. the complexity of the network. The neuron model used was complex enough to replicate key features found in real neurons, including adaptation, bursting, depolarization blockade, and voltage-sensitive NMDA conductance (Lytton and Stewart, 2005, 2006; Lytton and Omurtag, 2007; Lytton et al., 2008a,b; Neymotin et al., 2011), yet was simple enough to connect into large networks, allowing the model to capture multiscale dynamics. Each cell had a membrane voltage state variable (*V*_*m*_), with a baseline value determined by a resting membrane potential parameter (*V*_*RMP*_, set at either −63 or −65 mV depending on cell type). This membrane voltage is updated based on one of three events: synaptic input, threshold spike generation, and refractory period. Synaptic inputs are modeled using reversal potentials, time constants and delays specific to each synapse type: AMPA, NMDA, and GABA_*A*_. In addition to spikes generated by cells in the model, subthreshold Poisson-distributed spike inputs to synapses were used to provide ongoing activity, representing inputs from other neurons not explicitly simulated. Further details, including mathematical equations and parameter values, can be found in previously published papers (Neymotin et al., 2011, 2013; Chadderdon et al., 2012) or in the Supplementary Material section. The full model is publicly available via ModelDB (http://modeldb.yale.edu/183014), including the compiled virtual arm module.

The present neuronal network is used to describe the dynamics of major elements in the sensorimotor learning loop (Wolpert et al., 2011): sensory input, internal processing and motor output. We therefore instantiate three different neural populations labeled proprioceptive (P), somatosensory (S), and motor (M).

The P population consisted of 192 units implemented as spike generators (NetStims in NEURON), which receive input from the virtual musculoskeletal arm, and project to the S population. Units were divided into four subpopulations, each encoding the average muscle length of a specific group of muscle: shoulder extensors (Pse), shoulder flexors (Psf), elbow extensors (Pee), and elbow flexors (Pef). Within each P subpopulation, each individual cell was tuned to fire strongly (100 Hz) to a specific range of muscle lengths. This range was calculated by dividing the total range of possible lengths (23 cm –3 cm = 20 cm) by the number of units in each P subpopulation (48), and allowing for a 50% overlap in adjacent units. Thus, this represented a type of population coding where the width of the tuning curve (square wave) of each unit was 8.33 mm, and the distance between each curve was 4.17 mm.

The exact encoding of proprioceptive information in the sensoriomotor system is not yet known. However, it has been established that muscle spindles convey muscle length information (Francis, 2009), and evidence suggests that this information is combined via population coding to form a representation of limb kinematics (Bergenheim et al., 2000; Roll et al., 2004). Our model provides a simplified implementation of these features.

**Figure 2:**
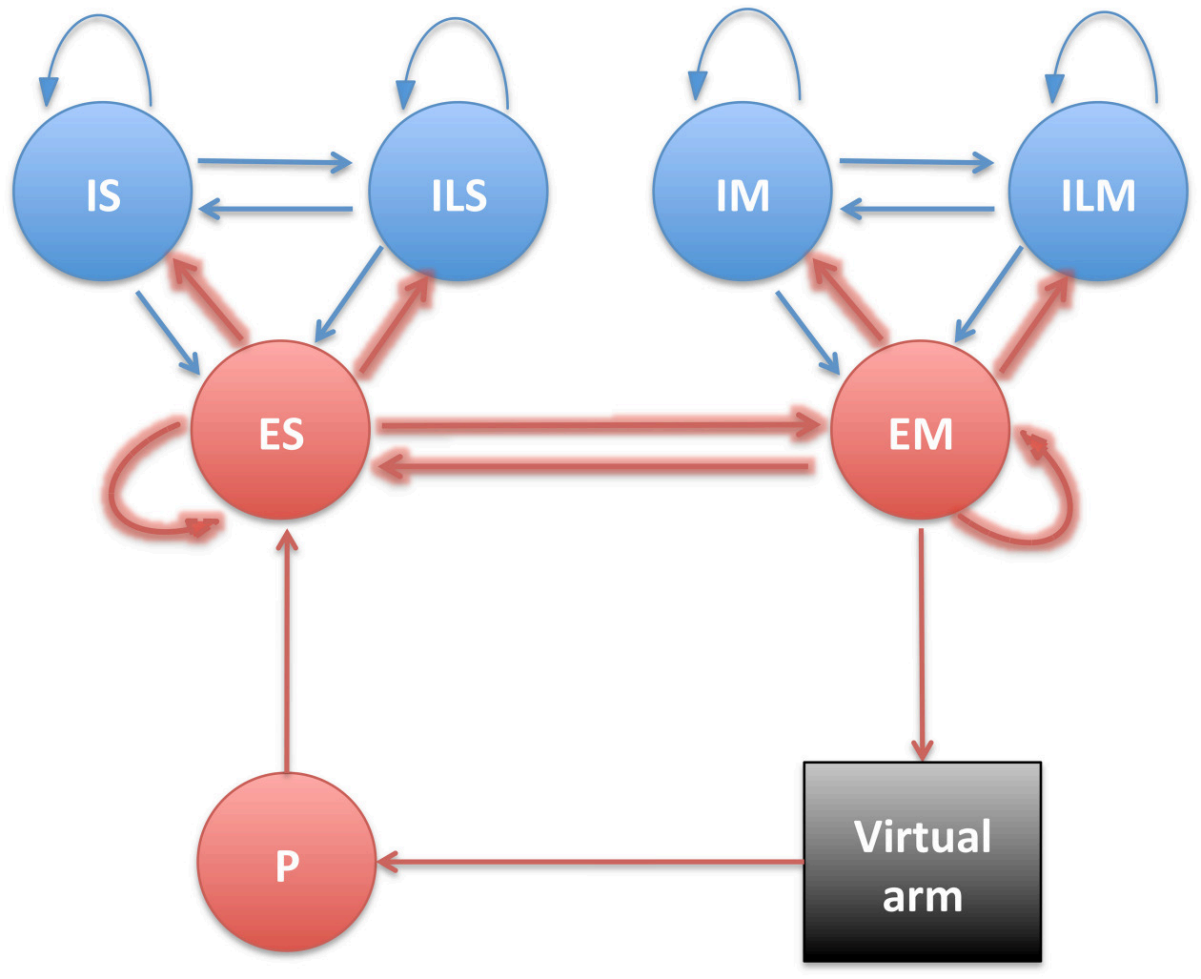
**Diagram showing the different neural populations with arrows indicating connections (plastic connections are shown as thicker arrows)**.

S and M populations were each comprised of 192 excitatory cells (ES and EM), 44 fast-spiking inhibitory cells (IS and IM), and 20 low-threshold inhibitory cells (ILS and ILM), with recurrent connectivity between the E and I cells of each population (Figure 2). Detailed connectivity parameters can be found in the Supplementary Material section. The number of excitatory to inhibitory cells within an area was selected to keep a 4:1 ratio, to approximate the ratios in neocortex. ES cells received fixed weight afferent connections from units in all P subpopulations, such that they were capable of representing the conjunction of multiple muscle lengths, and therefore of the full arm posture. The ES cells then sent plastic weight connections to the EM cells, which were divided into four subpopulations, one for each muscle group, analogously to the P population: EMse, EMsf, EMee, EMef. The muscle excitation to each muscle group was calculated by adding the number of spikes of the corresponding subpopulation over a short time sliding window, and dividing by a normalizing constant. The network effectively performed a mapping between limb state, as measured by muscle length, and the muscle excitation required for driving each muscle.

The spiking network simulations were run in NEURON 7.3 (Hines and Carnevale, 2001; Carnevale and Hines, 2006) on a Linux workstation with 24 Intel Xeon 2.7 GHz cores and on a High-Performance Computing system with 512 AMD Opteron 2.6 Ghz cores.

### 2.3. Learning rule

In order to implement a biologically-plausible reinforcement learning rule, the system was formulated in terms of an Actor-Critic framework, where the Actor generates actions that affect the environment based on current perception (control policy), and the Critic provides a reward or punisher signal to the Actor (value function). In our system, the spiking neuronal network provided an Actor which mapped muscle lengths (perception) to muscle excitations (action). The environment consisted of the virtual musculoskeletal arm and a fixed target. The Critic was provided as a global reward/punisher signal which modulated plasticity changes. To determine the Critic's signal, the difference between the hand's location and the target was calculated for the last two time steps (10 ms interval), such that if the hand was getting closer from the target, a reward signal would be sent to the Actor; and if the hand was getting farther, a punisher signal would be sent instead.

Connections between cells of different populations were established probabilistically based on connection densities and initial synaptic weight parameters set for each pair of pre- and postsynaptic cell types. Plasticity was present within the S and M unit populations and between them in both directions, and was implemented using a reward-modulated spike-timing dependent plasticity (STDP) rule. The credit-assignment problem was handled using a synapse-specific memory, called an eligibility trace (Izhikevich, 2007). The trace was only imprinted on the eligibility-tagged synapses when a global modulatory signal was received from the Critic. Synaptic weights were increased, long-term potentiation (LTP), or decreased, long-term depression (LTD), depending on whether the Critic's modulatory signal was positive (reward) or negative (punishment). See the Supplementary Material section for further details on the learning rule, including equations and parameter values.

One important component of reinforcement learning was exploratory behaviors, which can be implemented using “motor babbling” (DeWolf and Eliasmith, 2011). Motor commands are randomly selected, and their outcomes and associated rewards are used for learning. We implemented motor babbling by increasing the background noise of subpopulations controlling the different muscle groups for short periods of time (max 1.5 s). By increasing the excitation of a single muscle group at a time, we ensured that the virtual arm performed substantial movements. Both the muscle group being stimulated and the duration of stimulation were selected randomly. These movements, coupled with reward-modulated synaptic plasticity, enabled the system to learn the appropriate mapping between muscle lengths and muscle excitations required to reach the target.

We initially checked that the system could be trained to reach a target using the proprioceptive information from the virtual arm. We chose two different targets (left and bottom), located at 15 cm from the starting position. Reaching was considered successful if the hand was able to reach the target area, defined as a 4 cm radius circumference around the target center. This task is comparable to the classical center-out reaching task employed experimentally in humans (Demandt et al., 2012; Flint et al., 2012) and monkeys (Hatsopoulos et al., 2004; Sanchez et al., 2011). The network was trained to reach one target at a time, by enforcing random exploratory movements of the arm and modifying the network synaptic weights via reinforcement learning. The starting position of the arm in both phases corresponds to a shoulder angle of 35° (0.62 rad) and an elbow angle of 88°(1.53 rad), consistent with the arm's natural resting position. The minimum and maximum angles were −10° and 110° for the shoulder joint, and 0° and 140° for the elbow joint. Robot arm testing was performed with a maximum elbow flexion angle of 135°; and this was later set to 140° to increase the range of movement when reaching to the bottom target.

The model does not include any explicit mechanisms to stop the arm, so the duration of the testing trials was limited to one second. Holding the position after reaching could be implemented by using an external control signal that accounted for the contribution of non-modeled regions, such as premotor cortex, thalamus or basal ganglia, to the initiation and stopping of movement, similar to that employed in other models (DeWolf and Eliasmith, 2011; Sussillo et al., 2015).

Network and training metaparameters, such as the learning rate or the motor command normalizing constant, were optimized using evolutionary algorithms to minimize the overall trajectory distance to target. See Supplementary Material section for more details on the optimization method and a complete list of parameters optimized.

### 2.4. Interface between neuronal model and virtual arm

The virtual musculoskeletal arm runs as a standalone C++ executable. This had to be integrated with the interpreted NEURON model in a closed loop. Because the musculoskeletal arm code could not be embedded in the simulator, we employed inter-process communication methods. This had the additional advantage of producing a flexible system where the neuronal network and virtual arm simulations could either be executed on the same machine or on separate machines for greater speed.

In our previous work, we had implemented external interfaces for NEURON using UDP network communication (Dura-Bernal et al., 2014a), which however proved limiting in terms of communication robustness. We were able to improve upon this in the current design by using pipes.

**Figure 3:**
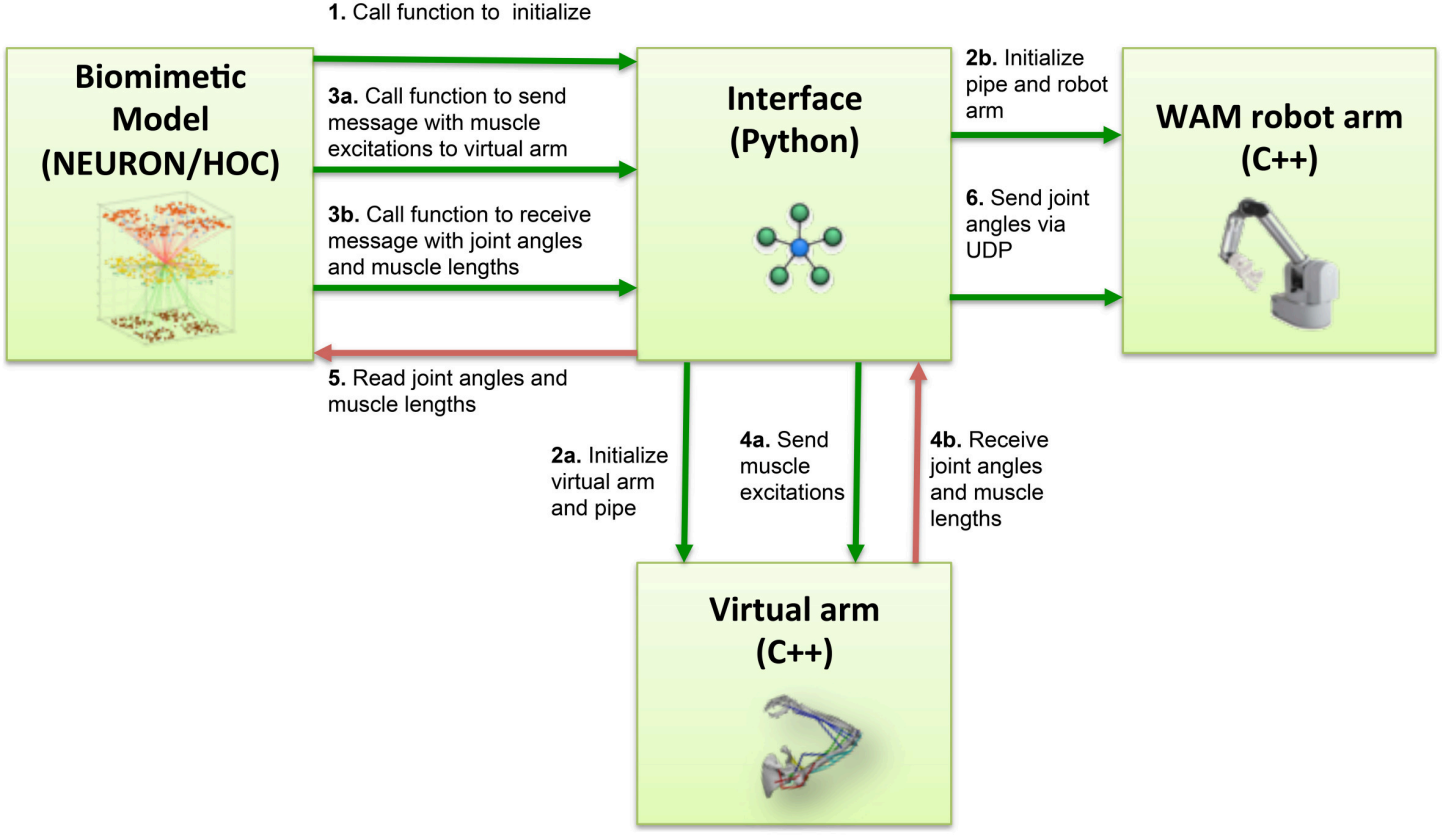
**Block diagram of the information flow in the system**. Blocks represent the four main software components of the system: NEURON biomimetic model, C++ virtual arm, C++ robot arm code, and the Python interface, responsible for linking all the previous. Arrows represent the information flow between software components, and numbers indicate the order in which these occur.

Several steps were involved in the pipes interfacing process (Figure 3). During NEURON initialization, the Python subprocess function ran the virtual arm executable and enabled the input and output pipes. Subsequently, the neuronal model sent a message to the virtual arm at an update interval corresponding to 10 ms of simulated time, this being 10 iterations of the arm simulation (timestep 1 ms). This message contained the excitation values for each muscle group, calculated from the corresponding EM subpopulation firing rate. At the same time, the spiking model received from the virtual arm a message containing the joint angles (used to calculate the error during reinforcement learning), and a separate message with muscle lengths (used to update the proprioceptive population).

On the virtual arm side, three custom-developed event handlers were executed at every update interval, implementing the following functions: set the muscle excitations, output the muscle lengths, output the arm joint angles. Since the virtual arm code was executed as a subprocess, communication with the NEURON model was direct; the event handlers simply had to read the muscle excitations from the stdin stream, and write the joint angles and muscle lengths to the stdout stream.

### 2.5. Interface between neuronal model and robotic arm

Our previous interface between neuronal model and robot arm utilized a simple 2 degree-of-freedom virtual arm (Dura-Bernal et al., 2014a). We now advanced this model by utilizing the virtual musculoskeletal arm as the intermediary in order to give the robot arm some of the additional effector constraints that the musculoskeletal platform provides, as well as the potential for improved feedback.

We again employed the Whole Arm Manipulator (WAM) robot developed by Barrett Technology (Barrett, 2012; Dura-Bernal et al., 2014a). The WAM internal computer, embedded in the base of the WAM arm, controlled the robot movements at a rate of 500 Hz, by sending motor torques to the WAM arm motors and receiving as feedback the motor positions. An open-source C++ library, Libbarrett^1^, provided high-level functions to control the WAM arm from the internal computer. The WAM included a small router that allowed an external computer to connect to the internal WAM computer, to both remotely run code in the internal computer to provide two-way communication in real-time between internal and external computers. Here, Barrett's WAM robot arm was driven in real time using the joint angles provided by the virtual musculoskeletal arm, which was in turn driven by the spiking neural network.

**Figure 4:**
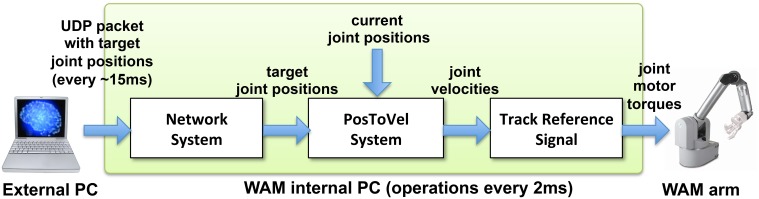
**Diagram of WAM robot arm control method**. The external processor sends UDP packets with the target joint positions approximately every 15 ms. The Libbarrett code running synchronously at 500 Hz in the internal WAM CPU, extracts the target joint angular position vectors (Network System); converts them into joint angular velocities (PosToVel System); and generates the required joint torques for each motor using the TrackReferenceSignal function.

Our NEURON-based Python interface was used to initialize a UDP socket and send UDP packets containing the joint angles received from the virtual arm to the WAM robot's internal computer (Figure 3). The internal robot computer received UDP packets with the desired joint angles and update the arm position every update interval. We implemented two custom systems as Libbarrett classes (Figure 4) that were executed in real time (500 Hz) by the WAM internal computer. The first one was the *Network System*, responsible for initializing and reading incoming packets from the UDP socket, and outputting a vector of joint angular positions to the arm. The *PosToVel System* then used the target and the current joint angular positions to calculate the required joint angular velocities every 2 ms. Overall, these two systems provided the transform from asynchronous incoming network packets with desired joint positions to the required synchronous robot joint torques.

## 3. Results

### 3.1. Encoding of virtual arm in the biomimetic spiking model

**Figure 5:**
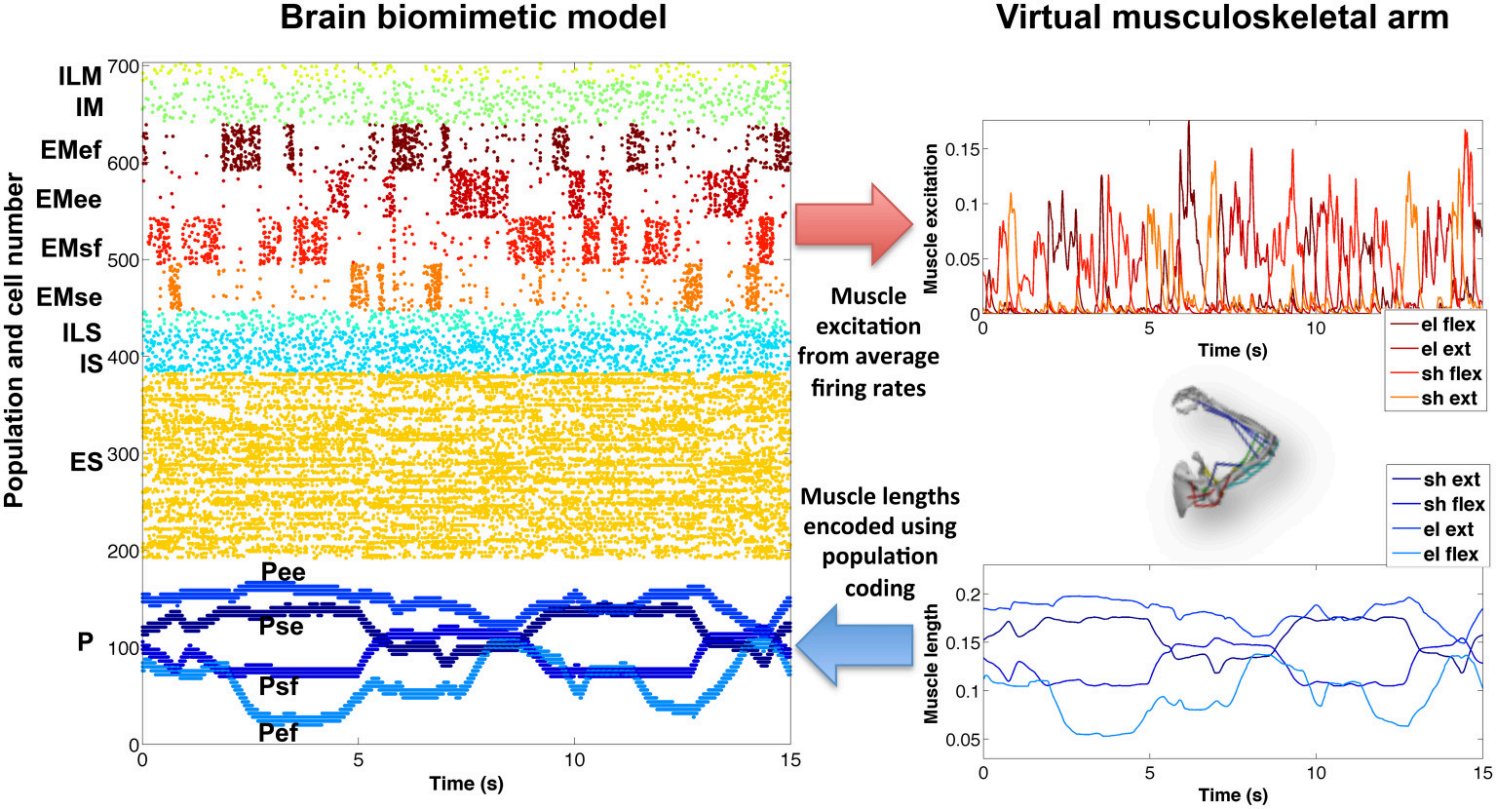
**Encoding of virtual arm muscle excitations (top right) and lengths (bottom right) in the biomimetic spiking model firing patterns (raster plot on the left)**. For comparison purposes, the same colors have been used to represent the spikes corresponding to the excitations (or lengths) of each muscle group. Note that P cells are ordered by muscle length, such that cells belonging to each muscle group subpopulation are spaced (in steps of 4) across the full P population, e.g., *P*_*sf*_ = cells 0, 4, 8, …, 188.

To demonstrate successful communication between the virtual musculoskeletal arm and the cortical spiking model, we compared the network's firing activity (raster plot) with the virtual arm muscle excitations and lengths (Figure 5), for a 15-s training period with random exploratory movements. The excitatory motor (EM) population is divided into four subpopulations, one for each muscle group. The normalized average firing rate of each subpopulation, calculated over a sliding window of 80 ms, corresponded closely to the input excitation of each muscle group. In this example, the firing patterns of each subpopulation were clearly segregated (no coactivation) because they were driven by artificially external noise aimed at activating one muscle group at a time, in order to enforce exploratory movements during training.

Similarly, the proprioceptive (P) population encoded the average muscle length of each muscle group in four different subpopulations. In this case, we employed a type of population coding, where each neuron was tuned to a small subset of muscle lengths. The P firing patterns accurately represented the virtual arm muscle lengths over time. For symmetry breaking and to make the spiking patterns more realistic, a certain amount of noise could be added to the proprioceptive neurons (omitted in Figure 5, for clarity).

The new arm model showed improved biological correspondence in terms of the realism of what was being controlled and represented by the network. The effectors in the new model provide muscle activation rather than a direct control of joint angle. Afferents now measure muscle length, a signal that comes from muscle spindle proprioceptors embedded in muscle, rather than normalized joint angle. This allowed the new model to capture the non-linear relations from joint angles to muscle lengths and from spiking to muscle excitation to force to new joint angles (previously, firing rate had a linear effect on joint angle).

### 3.2. Virtual arm trajectories and forces

**Figure 6:**
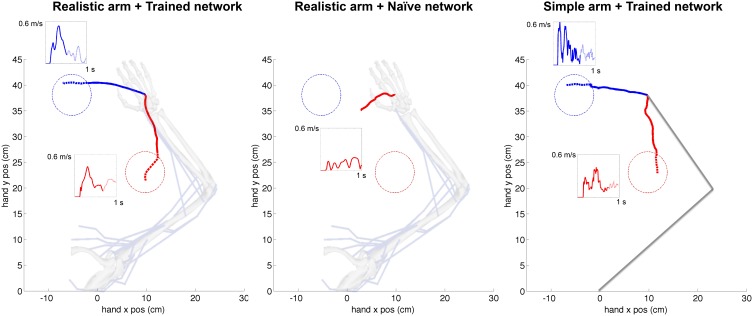
**Representative example of cartesian trajectories and velocity profiles of virtual arm during a reaching task to two targets for realistic arm and trained network (left), realistic arm and naive network (center), and simple arm and trained network (right)**. Trained networks employed STDP-based reinforcement learning to adapt its synaptic weights to drive the virtual arm to each of these two targets. The naive network drives the arm using the initial random weights and therefore produces random movements independent of the target location. The hand trajectories of the realistic musculoskeletal arm are smoother than those of the simple arm, and show velocity profiles consistent with physiological movement. The initial reaching trajectory from starting point to target area is shown as a solid line, whereas the final part of the trajectory within the target area is shown as a dotted line. The starting configuration of the simple and musculoskeletal arm is shown in the background.

The cortical spiking network was trained using reinforcement learning based on the joint angles fed back from the virtual arm, demonstrating this external input information can be successfully used for training purposes. A representative example of the cartesian trajectories and velocity profiles of the virtual hand reaching to two targets (left and bottom) is shown in Figure 6. Compared to the naive network, which performed random movements independent of target location, the network trained for 360 s of simulated-time was able to reach the target area (4 cm radius) within 1 s. Compared to a trained network but using the simple arm model, the realistic musculoskeletal arm model showed smoother spatial and temporal profiles, closer to those performed by primates during fast reaching movements (Shadmehr and Mussa-Ivaldi, 1994; Berger and d'Avella, 2014). The same neural encoding, training and optimization methods were used for the simple arm model and the realistic musculoskeletal arm model: 29/30 (left) and 26/30 (bottom) trained networks reached the target area using the musculoskeletal arm; and 28/30 (left) and 27/30 (bottom) trained networks successfully reached the target area using the simple arm.

The average velocity of the musculoskeletal arm, across successful trajectories, was higher between movement onset and reaching the target area (left: mean = 0.327 m/s, *SD* = 0.051 m/s; bottom: mean = 0.208 m/s, *SD* = 0.039 m/s), than after reaching the target area (left: mean = 0.167 m/s, *SD* = 0.030 m/s; bottom: mean = 0.184 m/s, *SD* = 0.049 m/s). Even though the model did not implement explicit stopping mechanisms, this velocity decrease, coupled with changes in movement direction, contributed to the arm remaining within or close to the target area: the mean distance to target center, from the time the arm first reached the target area until the end of the trial, was 3.87 cm (*SD* = 0.78 cm) and 3.20 cm (*SD* = 0.69 cm), for the left and bottom targets respectively. The mean time that the arm remained within the target area was 295 ms (*SD* = 12 ms) for the left target, and 202 ms (*SD* = 10 ms) for the bottom target. These results share similarities with experimental center-out reaching tasks in primates, where the subject is required to stay within the target area for a short period of time, known as hold time (usually 200–500 ms; Hatsopoulos et al., 2004; Flint et al., 2012), after which the trial is considered finalized.

The smoothness of trajectories was quantified by calculating a dimensionless measure of jerk (rate of change of acceleration). The reason to a dimensionless measure is that smoothness is an aspect of movement quality that should be independent of speed and distance. The dimensionless jerk measure (Hogan and Sternad, 2009) is calculated as $\left(\int_{t1}^{t2}\dddot{x}{\left(t\right)}^{2}dt\right)\cdot D^{3}/v_{mean}^{2}$, where $\overset{\mathrm{...}}{x}\left(t\right)$ is the jerk at time *t*, *D* = *t*_2_−*t*_1_ is trajectory duration, and *v*_*mean*_ is the mean trajectory speed. It reflects changes of movement shape considered common departures from smoothness, including multiple speed peaks or periods of arrest, but is independent of amplitude and duration. For example, this measure has been previously used to compare the movement smoothness of patients with Parkinson's disease and age-matched unimpaired subjects (Teulings et al., 1997).

**Figure 7:**
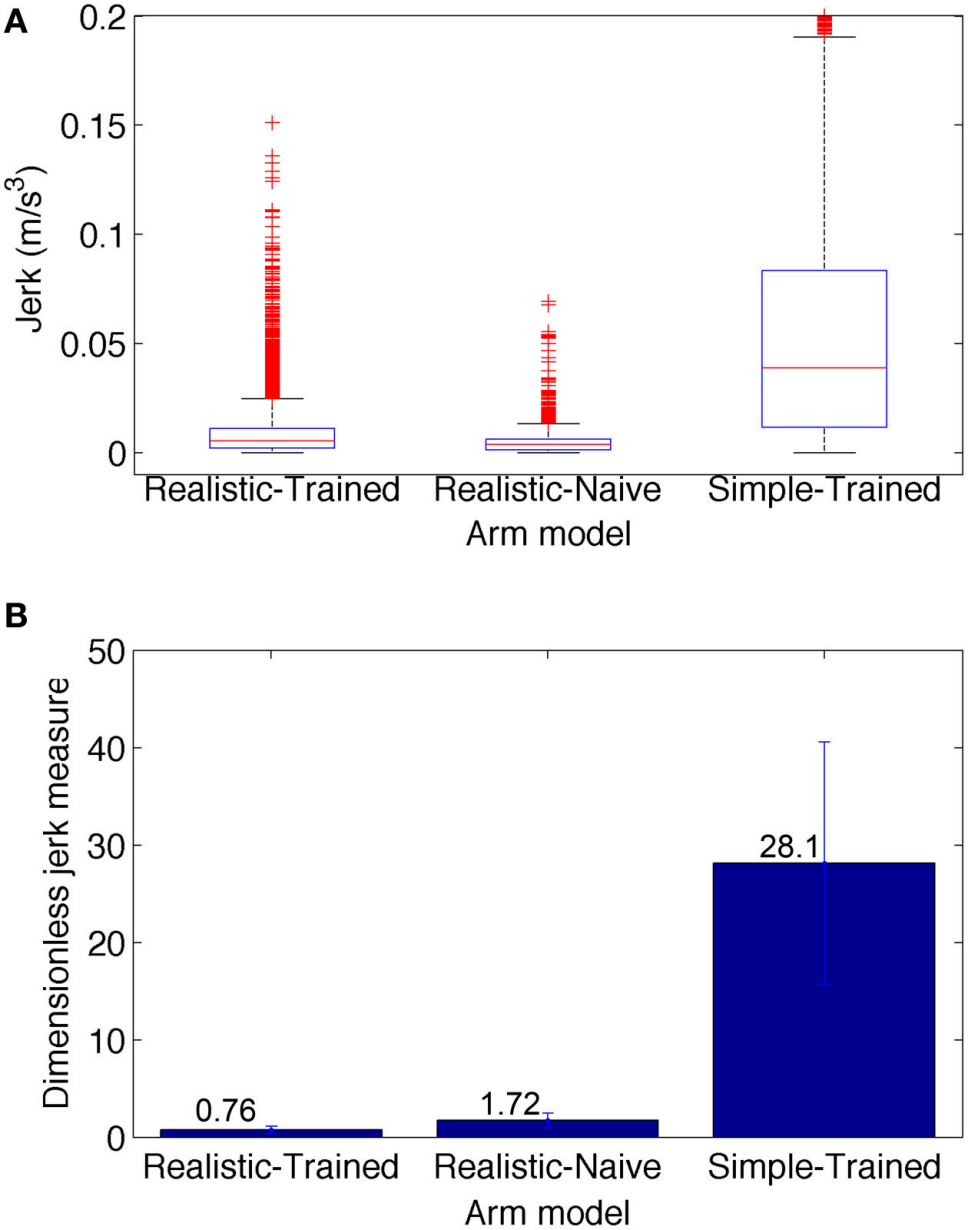
**Jerk-based comparison of smoothness for the three conditions depicted in Figure 6: realistic arm and trained network (left), realistic arm and naive network (center), and simple arm and trained network (right)**. **(A)** Boxplot statistics of the jerk (rate of change of acceleration). **(B)** Dimensionless jerk measure, which quantifies jerk but is independent of trajectory amplitude and duration. The dimensionless jerk measure was lower for the realistic musculoskeletal arm as compared to the simple arm, both in the naive and trained networks. Jerk was calculated based on reaching movements obtained from the best 30 metaparameter configurations for each case. Number of points = 5700 for each condition; number of outliers (red crosses) = 481 (Realistic-Trained), 581 (Realistic-Naive), and 297 (Simple-Trained; not shown for clarity).

To compare the smoothness of the three conditions depicted in Figure 6, we calculated the jerk (Figure 7A) and the dimensionless jerk measure (Figure 7B) for each trajectory. The mean dimensionless jerk measure was significantly lower for the realistic arm as compared to the simple arm (28.1), both in the trained (0.76), and naive networks (1.72). The similar jerk values of naive and trained networks suggests the musculoskeletal arm generates smoother movements than the simple arm for any biologically reasonable input. Naive network trajectories were shorter (lower velocities) than trained network trajectories, due to lower overall synaptic weights. This was reflected in the slightly lower median jerk values (Figure 7A) of the naive network, but not in the dimensionless jerk measure (Figure 7B), which is independent of speed. Jerk was calculated using data from 30 different reaching movements for each case, generated by the top metaparameter configurations.

**Figure 8:**
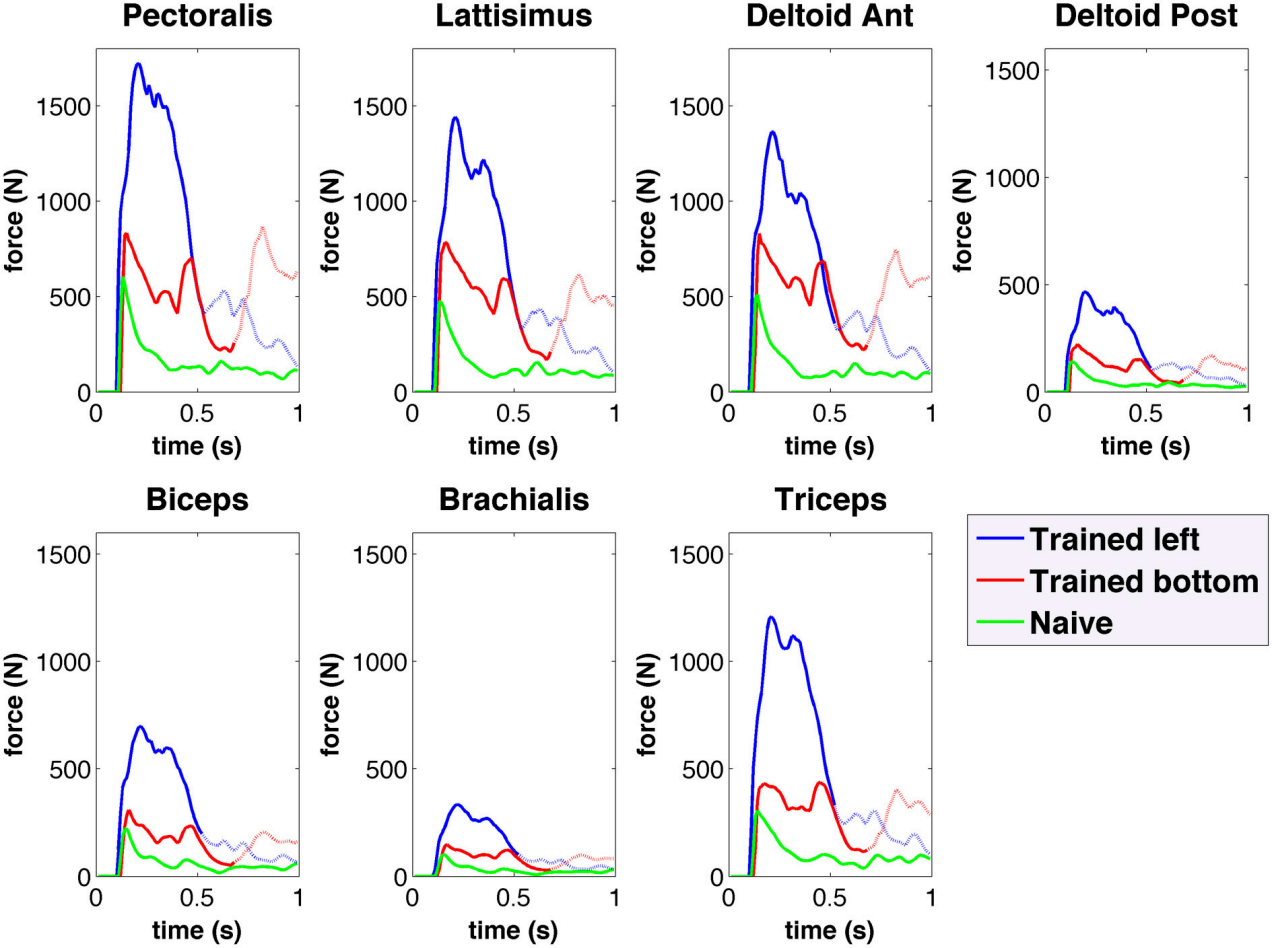
**Forces obtained from the spiking network driven virtual arm muscles during center-out reaching to left (blue) and bottom (red) targets for the trained network, and for the naive network (green)**. Forces are shown for seven of the main muscles involved in reaching. The trained network muscle forces are higher than those of the naive network, and show differentiated activation for each target.

The musculoskeletal arm model provides access to detailed information, including muscle activation, length, active and passive forces, fatigue, tendon length, tendon force, and pennation angle. As an example, Figure 8 shows the active muscle forces corresponding to the reaching trajectories shown in Figure 6. Compared to the naive network, forces in the trained network are higher and exhibit distinct activation patterns for each target. The resulting arm trajectories are a consequence of the complex balance of muscle forces, such that relative amplitude differences between antagonistic muscle forces can determine the final arm direction. Having access to the detailed muscle information allows us to correlate it with the neural data and extract conclusions to improve the system. For example, compared to human experimental data (Berger and d'Avella, 2014), the model showed excessive coactivation of the muscles and high correlation to the output motor population. This was possibly due to lack of selectivity during learning, or lack of inhibitory mechanisms, such as those present in the spinal cord (Alstermark and Isa, 2012).

### 3.3. Robot arm trajectories

**Figure 9:**
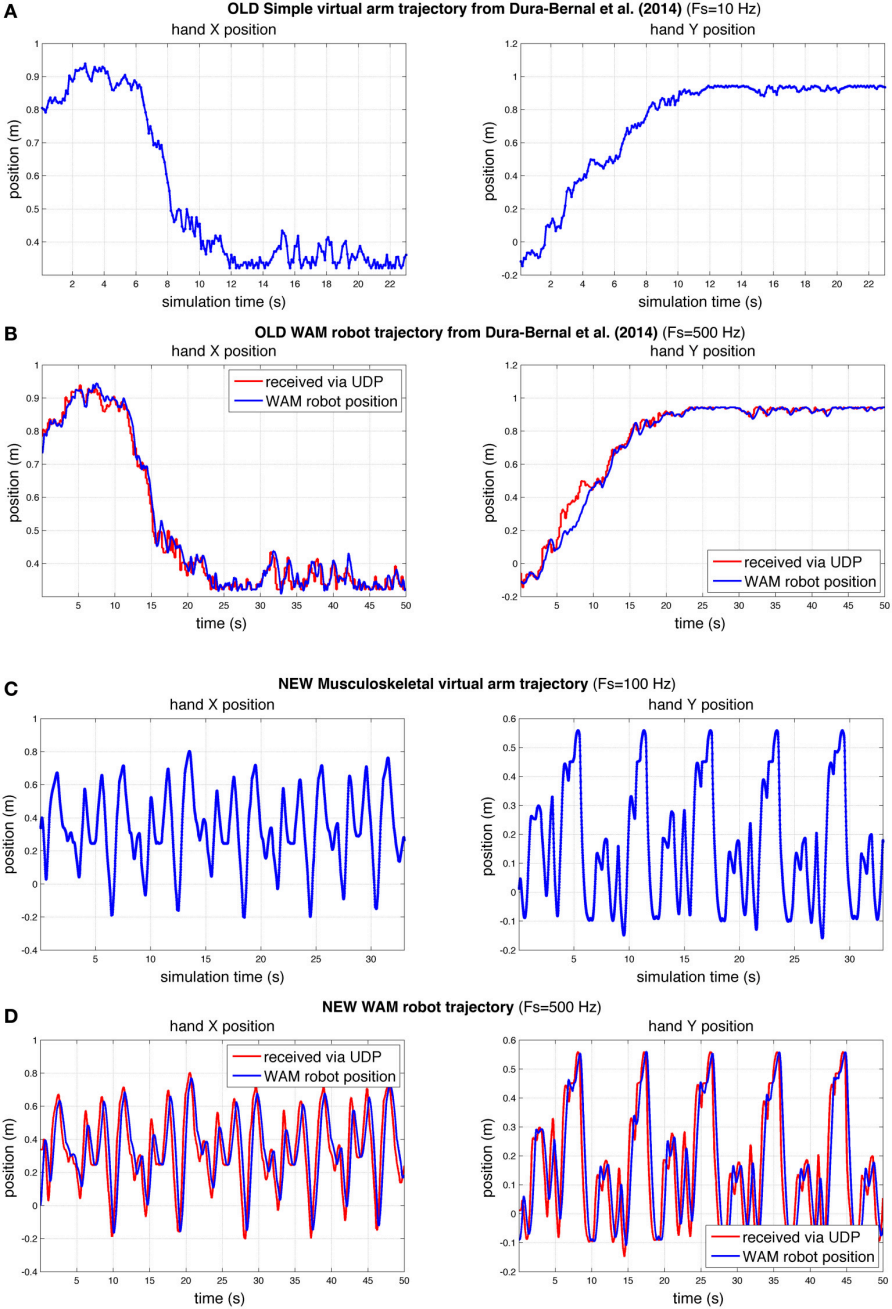
**Comparison of hand X and Y position of virtual and robot arm trajectories in our previous (Dura-Bernal et al., 2014a) and current papers**. Compared to our previous paper **(A,B)**, here the robot arm was able to follow a more challenging trajectory in real time with a higher degree of accuracy **(C,D)**. The rate of update from the spiking model to the simple **(A)** and musculoskeletal **(C)** virtual arms, was 10 and 100 Hz of simulated time, respectively. WAM robot arm update rate **(B,D)** was 500 Hz; blue line represents actual robot position, whereas red line shows virtual arm trajectory received at robot arm from spiking model via network UDP.

To demonstrate that our interface was able to make the WAM robotic arm follow the virtual arm movements, we compared the virtual and robot arm trajectories over time. We also compared the trajectories in our previous (Dura-Bernal et al., 2014a) and current papers (Figure 9). Our previous paper employed a simple arm model updated every 100 ms of simulated time (Figure 9A) to drive the robot (Figure 9B). In this paper we make use of a musculoskeletal arm model updated every 10 ms of simulated time (Figure 9C) and an improved interface to drive the robot (Figure 9D). Direct comparison of the old and new robot systems is not possible since the old system was trained to perform a different reaching task. However, to compare performance, we calculated accuracy and smoothness measures, independent of distance traveled, for old and new robot trajectories of the same duration (50 s).

The results obtained for the new musculoskeletal-based system (Figure 9D) show that, except for a small undershoot during the highest velocity peaks, the robot accurately followed the received trajectory. Both the trajectory sent by the biomimetic model via network UDP (red line) and the trajectory followed by the robot arm (blue line) were recorded at the WAM robot internal PC at a fixed real-time rate of 500 Hz, such that both signals shared the same time source. The mean absolute angle difference between the robot and virtual arm was 1.85° (*SD* = 2.19°) for the shoulder joint, and 1.74° (*SD* = 2.11°) for the elbow joint. Notably, these errors were calculated for a 50-s training trajectory, with enforced exploratory movements, where joint angles varied over 100° at angular velocities above 60°∕*s*. The angular error divided by total angular displacement was 0.00095 (shoulder) and 0.0012 (elbow), which are lower than the values obtained in our previous study: 0.0071 (shoulder) and 0.0026 (elbow). Note that the two robot trajectories compared had identical duration, but the new trajectory exhibited higher velocities and total displacement.

**Figure 10:**
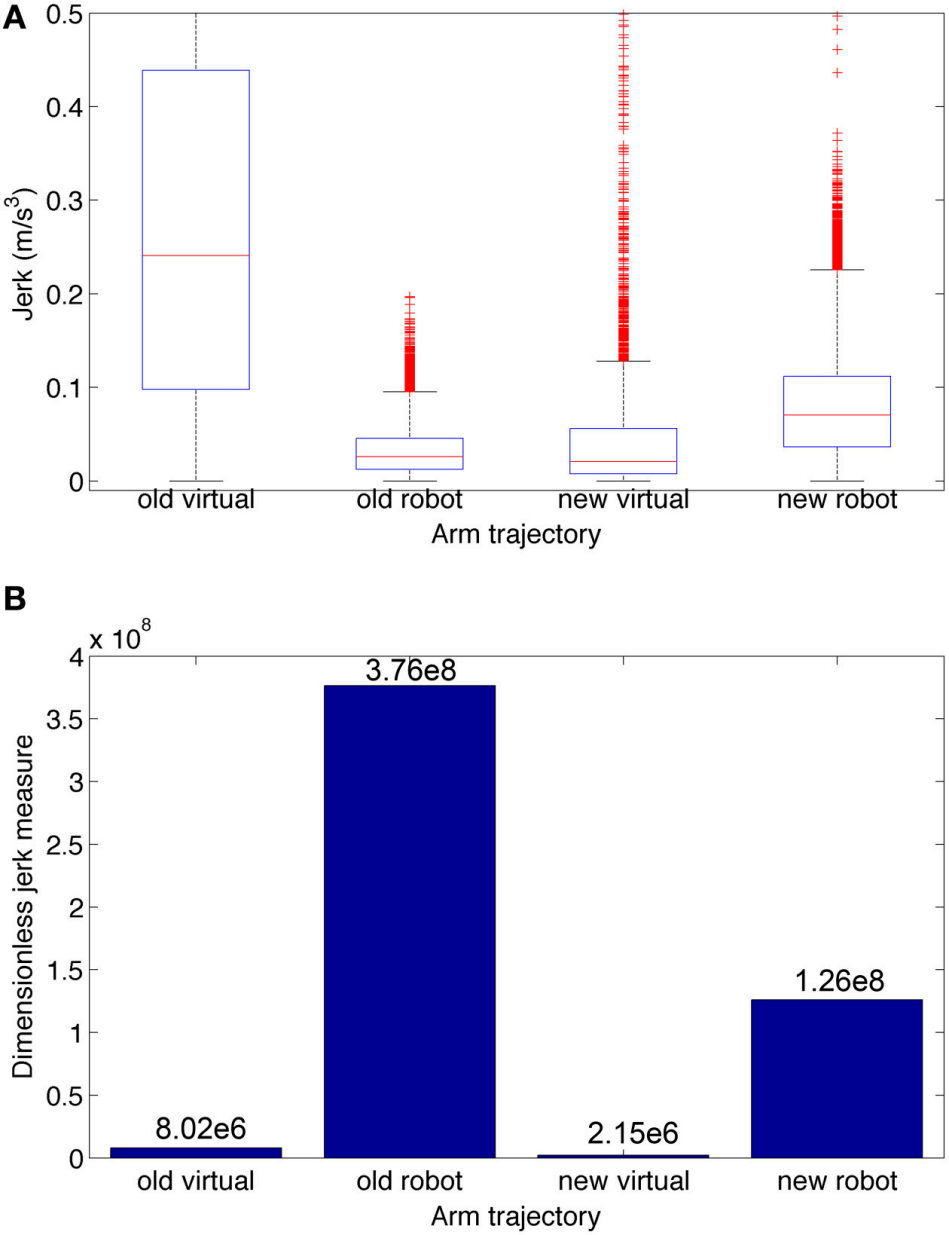
**Jerk-based comparison of smoothness for the four trajectories depicted in Figure 9: virtual and robot arm trajectories from the previous (“old”) and current (“new”) papers**. **(A)** Boxplot statistics of the jerk (rate of change of acceleration). **(B)** Dimensionless jerk measure, which quantifies jerk but is independent of trajectory amplitude and duration. The dimensionless jerk measure of the new virtual and robot arm trajectories was lower than those in our previous paper. Number of points/outliers = 227/11 (old virtual), 24997/585 (old robot), 3297/350 (new virtual), and 24997/610 (new robot).

To quantify the smoothness we calculated the jerk and the jerk-based dimensionless measure (Figure 10) for each of the trajectories in Figure 9. Based on the dimensionless jerk measure, both the virtual and robot arm trajectories in this study were smoother than in the previous one (Dura-Bernal et al., 2014a). These results should be evaluated cautiously given the different shapes of the trajectories compared. Nonetheless, they provide an estimate of smoothness, independent of amplitude and duration, which suggests the new musculoskeletal arm and robot control system contribute to increased smoothness in the robot trajectories.

The mean interval between incoming network packets to the robot arm was 15.09 ms (*SD* = 5.65 ms). This means the full system, including neural simulation, virtual arm model and robot arm, was updating at approximately 67 Hz, very close to the cortical spiking model theoretical output rate of 100 Hz (packets are sent every 10 ms of simulated time). The maximum interval was 46 ms, but 95% of the packets arrived within 6 and 26 ms. The interval variability resulted mainly from differences in the computational requirements during the simulation, for example, longer computation for time steps when synaptic weights were being updated.

The mean delay between the virtual arm (red line) and robot arm (blue line; Figure 9D) was 312 ms (*SD* = 127.64 ms), obtained by calculating the shift that maximized the cross-correlation between the trajectories. This delay results from the robot arm internal control system, which converts the incoming target joint angles to the required joint torques.

**Figure 11:**
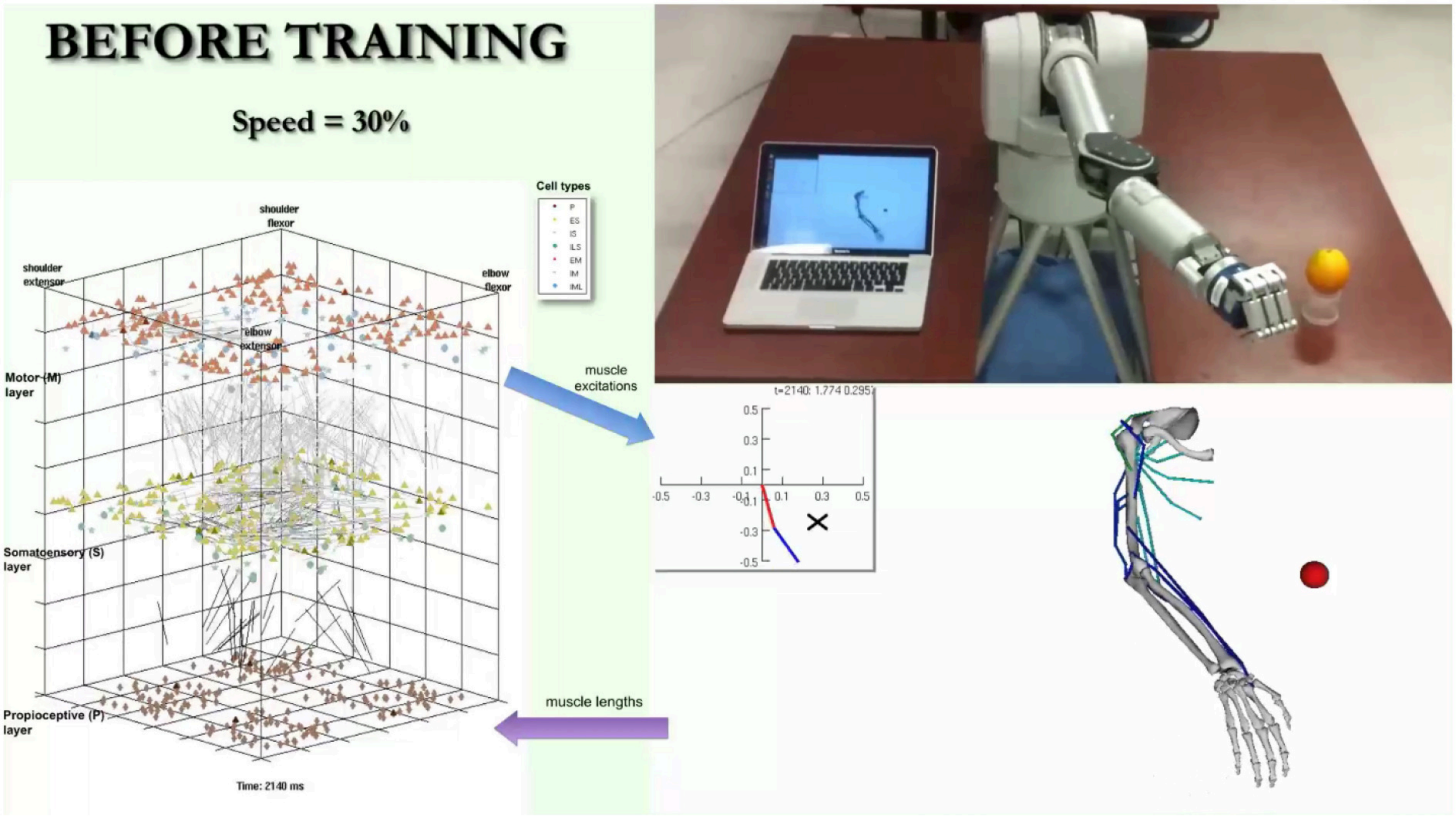
**Screenshot of video illustrating system**. The video simultaneously shows the biomimetic spiking model activity, virtual arm, and robotic arm, before, during, and after training to reach a target. It illustrates the interactions between the spiking model and virtual arm, and how the robotic arm closely follows the virtual arm trajectories. The video is included in the Supplementary Material and available for download from: http://www.neurosimlab.org/salvadord/biomimetic-vid.mp4.

A video is included in the Supplementary Material section in order to provide a real-life demonstration of the full working system. A screenshot of the video (Figure 11) shows the almost simultaneous movements of the virtual and robot arms (driven by the spiking model), before, during and after training to reach a target. The biomimetic spiking model is shown on the left-hand side, together with the spiking activity corresponding to the arm movements. The final grasping behavior was hard-coded for illustrative purposes. This demonstrates the overall system architecture, set up and dynamic interactions among the various components.

## 4. Discussion

We show here that a biomimetic neuronal network can control a robotic arm more realistically through interposition of a realistic arm simulation between the spiking movement generator and the robot. This enabled us to overcome several shortcomings seen in our prior studies (Dura-Bernal et al., 2014a). Our new musculoskeletal arm model included biophysical and mechanical properties of muscles, tendons and bones, rendering a complex non-linear dynamical system controlled via muscle excitations, and feeding back muscle lengths. This complex arm model provided biomimetic musculoskeletal dynamics that increased the smoothness of reaching trajectories to target compared to the prior highly-simplified intermediary arm model, where the arm was simply characterized by two joint angles which responded linearly to firing rate (Chadderdon et al., 2012; Neymotin et al., 2013). Including the realistic virtual arm in the sensorimotor loop linked the spatiotemporal scale of neural dynamics with that of movement, providing detailed access to all the elements in the system, ranging from individual spikes to muscle lengths (Figures 5, 6, 8). The musculoskeletal arm acted as a filter that smoothed the irregular spiking data by imposing realistic kinematic and dynamic constraints, such as elasticity, joint resistance and inertia, leading to smoother trajectories as compared to the previous simple arm (Figure 7). Additionally, the arm update rate was increased ~10-fold, reducing the robot incoming packet interval from 175 to 15 ms. This allowed the system to rely more on the virtual arm-generated trajectories and less on robot arm internal interpolation. The new system permitted smooth robot arm movement with low vibration even for challenging arm trajectories at ~ 4 × higher angular velocities (Figures 9, 10) than previously employed.

In future work we would like to continue adding realistic detail to the virtual arm and to the virtual arm control. It would be valuable to utilize individual muscles and even some individual muscle heads individually rather than dividing the muscles into four groups. This would also allow us to begin moving from the current 2D restricted movement into a 3D domain. We would also like to move forward by further evaluating antagonistic muscle coactivation and determining to what extent this can be learned and to what extent it should be provided as an additional biological constraint imposed at the spinal cord level. Finally, we are still calculating muscle excitation using simple thresholded linear readout from the spike rate. Employing a model with larger number of neurons or addition of a spinal cord population would allow us to control a larger number of muscle groups and encode more realistic transfer functions.

Future practical applications of our system required control of a real-world prosthetic arm, which presented different technical challenges to those of driving a virtual arm, and thus motivated the incorporation of a robotic arm interface. Although in this work proprioceptive feedback originated from the virtual arm, we previously showed that real-world position feedback from the robot arm could be incorporated into the network (Dura-Bernal et al., 2014a). This feature could be included in our current system by updating the virtual arm position based on the robot arm feedback. Another interesting extension would be to incorporate object grasping using tactile information from the robot fingers sensors. This would require reducing the current delay between the virtual arm and the robot.

A similar study developed a spiking model of the cerebellum and demonstrated real-time learning and control of a robot arm (Carrillo et al., 2008; Luque et al., 2011). The authors introduced a learning mechanism that produces a predictive corrective output which deals with the problem of proprioceptive feedback delays, and with the problem of antagonistic muscle coactivation. Future versions of our system could benefit from such an approach, since it currently experienced both of these issues.

Synthetic data generated by computational models has been previously used to aid in the development of neuroengineering applications such as brain-machine interfaces (BMI) and neural control via stimulation. For example, synthetic data was used to evaluate the performance of reinforcement learning-based BMIs (Mahmoudi et al., 2013; Dura-Bernal et al., 2014b; Prins et al., 2014; Marsh et al., 2015); and similar biomimetic models have been used to reproduce and evaluate the effects of microstimulation in primary somatosensory cortex (Song et al., 2013), propose a simple neuroprosthetic solution to restore information flow in cortex (Kerr et al., 2012), or test the ability of an adaptive inverse neurocontroller to repair a simulated motor lesion (Li et al., 2015; Dura-Bernal et al., under revision).

Combining brain and limb biomimetic models for the control of movement has potential for use in prostheses for neurorehabilitation (Fagg et al., 2007; Sartori et al., 2013), providing the ability to replace or repair lesioned brain regions (Sanchez et al., 2012; Carmena, 2013). In the future, extended versions of the *in silico* brain models presented here could act as a substitute motor cortex module and interact with real brain regions in patients with brain damage. We have previously demonstrated that spiking data recorded from macaque dorsal premotor cortex (PMd) can be fed in real-time to our biomimetic spiking model and modulate its output to potentially select a target to reach (Lee et al., 2014). The use of biomimetic systems, both on the neuronal and limb side, represents a novel approach that aims to utilize biological constraints to obtain more readily assimilated prosthetic system components, as compared to those provided by standard motor systems engineering.

### Conflict of interest statement

The authors declare that the research was conducted in the absence of any commercial or financial relationships that could be construed as a potential conflict of interest.

## Footnotes

^1^Barrett Technology Inc. Libbarrett C++ library.

## References

[B1] Almássy N. Edelman G. M. Sporns O. (1998). Behavioral constraints in the development of neuronal properties: a cortical model embedded in a real-world device. Cereb. Cortex 8, 346–361. 10.1093/cercor/8.4.346 9651130

[B2] Alstermark B. Isa T. (2012). Circuits for skilled reaching and grasping. Annu. Rev. Neurosci. 35, 559–578. 10.1146/annurev-neuro-062111-150527 22524789

[B3] Barrett (2012). WAM Training Documentation. Newton, MA:Barrett Technology Inc.

[B4] Bergenheim M. Ribot-Ciscar E. Roll J.-P. (2000). Proprioceptive population coding of two-dimensional limb movements in humans: I. muscle spindle feedback during spatially oriented movements. Exp. Brain Res. 134, 301–310. 10.1007/s002210000471 11045355

[B5] Berger D. J. d'Avella A. (2014). Effective force control by muscle synergies. Front. Comput. Neurosci. 8:46. 10.3389/fncom.2014.00046 24860489 PMC4029017

[B6] Carmena J. M. (2013). Advances in neuroprosthetic learning and control. PLoS Biol. 11:e1001561. 10.1371/journal.pbio.1001561 23700383 PMC3660243

[B7] Carnevale N. Hines M. (2006). The NEURON Book. New York, NY: Cambridge University Press.

[B8] Carrillo R. R. Ros E. Boucheny C. Coenen O. J.-M. D. (2008). A real-time spiking cerebellum model for learning robot control. Biosystems 94, 18–27. 10.1016/j.biosystems.2008.05.008 18616974

[B9] Chadderdon G. L. Neymotin S. A. Kerr C. C. Lytton W. W. (2012). Reinforcement learning of targeted movement in a spiking neuronal model of motor cortex. PLoS ONE 7:e47251. 10.1371/journal.pone.0047251 23094042 PMC3477154

[B10] Demandt E. Mehring C. Vogt K. Schulze-Bonhage A. Aertsen A. Ball T. (2012). Reaching movement onset- and end-related characteristics of eeg spectral power modulations. Front. Neurosci. 6:65. 10.3389/fnins.2012.00065 22586364 PMC3345572

[B11] DeWolf T. Eliasmith C. (2011). The neural optimal control hierarchy for motor control. J. Neural Eng. 8:065009. 10.1088/1741-2560/8/6/065009 22056418

[B12] Dura-Bernal S. Chadderdon G. L. Neymotin S. A. Francis J. T. Lytton W. W. (2014a). Towards a real-time interface between a biomimetic model of sensorimotor cortex and a robotic arm. Pattern Recognit. Lett. 36, 204–212. 10.1016/j.patrec.2013.05.019 PMC4689209 26709323

[B13] Dura-Bernal S. Prins N. Neymotin S. Prasad A. Sanchez J. Francis J. (2014b). Evaluating hebbian reinforcement learning bmi using an *in silico* brain model and a virtual musculoskeletal arm, in Neural Control of Movement. (Amsterdam).

[B14] Edelman G. M. (1987). Neural Darwinism: The Theory of Neuronal Group Selection. New York, NY: Basic Books. 10.1126/science.240.4860.1802 17842436

[B15] Fagg A. H. Hatsopoulos N. G. de Lafuente V. Moxon K. A. Nemati S. Rebesco J. M.. (2007). Biomimetic brain machine interfaces for the control of movement. J. Neurosci. 27, 11842–11846. 10.1523/JNEUROSCI.3516-07.2007 17978021 PMC2586067

[B16] Featherstone R. Orin D. (2000). Robot dynamics: equations and algorithms, in Robotics and Automation, Proceedings ICRA '00 IEEE International Conference on, Vol. 1 (San Francisco, CA), 826–834. Available online at: http://ieeexplore.ieee.org/stamp/stamp.jsp?tp=&arnumber=844153&isnumber=18235

[B17] Flint R. D. Lindberg E. W. Jordan L. R. Miller L. E. Slutzky M. W. (2012). Accurate decoding of reaching movements from field potentials in the absence of spikes. J. Neural Eng. 9:046006. 10.1088/1741-2560/9/4/046006 22733013 PMC3429374

[B18] Francis J. T. (2009). The neural representation of kinematics and dynamics in multiple brain regions: the use of force field reaching paradigms in the primate and rat, in Mechanosensitivity of the Nervous System, Mechanosensitivity in Cells and Tissues, Vol. 2, eds Kamkim A. Kiseleva I. (Netherlands: Springer), 215–247.

[B19] Hatsopoulos N. Joshi J. O'Leary J. G. (2004). Decoding continuous and discrete motor behaviors using motor and premotor cortical ensembles. J. Neurophysiol. 92, 1165–1174. 10.1152/jn.01245.2003 15277601

[B20] Hines M. L. Carnevale N. T. (2001). NEURON: a tool for neuroscientists. Neuroscientist 7, 123–135. 10.1177/107385840100700207 11496923

[B21] Hogan N. Sternad D. (2009). Sensitivity of smoothness measures to movement duration, amplitude, and arrests. J. Mot. Behav. 41, 529–534. 10.3200/35-09-004-RC 19892658 PMC3470860

[B22] Holzbaur K. R. Murray W. M. Delp S. L. (2005). A model of the upper extremity for simulating musculoskeletal surgery and analyzing neuromuscular control. Ann. Biomed. Eng. 33, 829–840. 10.1007/s10439-005-3320-7 16078622

[B23] Izhikevich E. M. (2007). Solving the distal reward problem through linkage of stdp and dopamine signaling. Cereb. Cortex 17, 2443–2452. 10.1093/cercor/bhl152 17220510

[B24] Kerr C. C. Neymotin S. A. Chadderdon G. L. Fietkiewicz C. T. Francis J. T. Lytton W. W. (2012). Electrostimulation as a prosthesis for repair of information flow in a computer model of neocortex. Neural Syst. Rehabil. Eng. IEEE Trans. 20, 153–160. 10.1109/TNSRE.2011.2178614 22180517

[B25] Lee G. Matsunaga A. Dura-Bernal S. Zhang W. Lytton W. Francis J. (2014). Towards real-time communication between *in vivo* neurophysiological data sources and simulator-based brain biomimetic models. J. Comput. Surg. 3:12 10.1186/s40244-014-0012-3 PMC4685709 26702394

[B26] Li K. Dura-Bernal S. Francis J. Lytton W. Principe J. (2015). Repairing lesions via kernel adaptive inverse control in a biomimetic model of sensorimotor cortex, in Neural Engineering (NER), 2015 7th International IEEE/EMBS Conference. (Montpellier).

[B27] Luque N. R. Garrido J. A. Carrillo R. R. Coenen O. J. Ros E. (2011). Cerebellar input configuration toward object model abstraction in manipulation tasks. Neural Netw. IEEE Trans. 22, 1321–1328. 10.1109/TNN.2011.2156809 21708499

[B28] Lytton W. W. Omurtag A. (2007). Tonic-clonic transitions in computer simulation. J. Clin. Neurophysiol. 24, 175–181. 10.1097/WNP.0b013e3180336fc0 17414973 PMC2633473

[B29] Lytton W. Stewart M. (2005). A rule-based firing model for neural networks. Int. J. Bioelectromagn. 7, 47–50. 10.1016/j.neucom.2005.12.066

[B30] Lytton W. W. Neymotin S. A. Hines M. L. (2008a). The virtual slice setup. J. Neurosci. Methods 171, 309–315. 10.1016/j.jneumeth.2008.03.005 18452996 PMC2398713

[B31] Lytton W. W. Omurtag A. Neymotin S. A. Hines M. L. (2008b). Just-in-time connectivity for large spiking networks. Neural Comput. 20, 2745–2756. 10.1162/neco.2008.10-07-622 18533821 PMC2562879

[B32] Lytton W. W. Stewart M. (2006). Rule-based firing for network simulations. Neurocomputing 69, 1160–1164. 10.1016/j.neucom.2005.12.066

[B33] Mahmoudi B. Pohlmeyer E. A. Prins N. W. Geng S. Sanchez J. C. (2013). Towards autonomous neuroprosthetic control using hebbian reinforcement learning. J. Neural Eng. 10:066005. 10.1088/1741-2560/10/6/066005 24100047

[B34] Marsh B. Tarigoppula A. Chen C. Francis J. T. (2015). Towards an autonomous brain machine interface: integrating sensorimotor reward modulation and reinforcement learning. J. Neurosci. 35, 7374–7387. 10.1523/JNEUROSCI.1802-14.2015 25972167 PMC6705437

[B35] Neymotin S. A. Chadderdon G. L. Kerr C. C. Francis J. T. Lytton W. W. (2013). Reinforcement learning of 2-joint virtual arm reaching in a computer model of sensorimotor cortex. Neural Comput. 25, 3263–3293. 10.1162/NECO/a/00521 24047323 PMC4291321

[B36] Neymotin S. A. Lee H. Park E. Fenton A. A. Lytton W. W. (2011). Emergence of physiological oscillation frequencies in a computer model of neocortex. Front. Comput. Neurosci. 5:19. 10.3389/fncom.2011.00019 21541305 PMC3082765

[B37] Prins N. W. Sanchez J. C. Prasad A. (2014). A confidence metric for using neurobiological feedback in actor-critic reinforcement learning based brain-machine interfaces. Front. Neurosci. 8:111. 10.3389/fnins.2014.00111 24904257 PMC4033619

[B38] Roll J.-P. Albert F. Ribot-Ciscar E. Bergenheim M. (2004). “Proprioceptive signature” of cursive writing in humans: a multi-population coding. Exp. Brain Res. 157, 359–368. 10.1007/s00221-004-1853-x 15007582

[B39] Sanchez J. Lytton W. Carmena J. Principe J. Fortes J. Barbour R.. (2012). Dynamically repairing and replacing neural networks: using hybrid computational and biological tools. IEEE Pulse 3, 57–59. 10.1109/MPUL.2011.2175640 22344954

[B40] Sanchez J. C. Tarigoppula A. Choi J. S. Marsh B. T. Chhatbar P. Y. Mahmoudi B. (2011). Control of a center-out reaching task using a reinforcement learning brain-machine interface, in Neural Engineering (NER), 2011 5th International IEEE/EMBS (Cancun: IEEE), 525–528.

[B41] Sartori M. Gizzi L. Lloyd D. G. Farina D. (2013). A musculoskeletal model of human locomotion driven by a low dimensional set of impulsive excitation primitives. Front. Comput Neurosci. 7:79. 10.3389/fncom.2013.00079 23805099 PMC3693080

[B42] Schutte L. M. Rodgers M. M. Zajac F. Glaser R. M. (1993). Improving the efficacy of electrical stimulation-induced leg cycle ergometry: an analysis based on a dynamic musculoskeletal model. Rehabil. Eng. IEEE Trans. 1, 109–125. 10.1109/86.242425

[B43] Shadmehr R. Mussa-Ivaldi F. A. (1994). Adaptive representation of dynamics during learning of a motor task. J. Neurosci. 14, 3208–3224. 8182467 10.1523/JNEUROSCI.14-05-03208.1994 PMC6577492

[B44] Song W. Kerr C. C. Lytton W. W. Francis J. T. (2013). Cortical plasticity induced by spike-triggered microstimulation in primate somatosensory cortex. PLoS ONE 8:e57453. 10.1371/journal.pone.0057453 23472086 PMC3589388

[B45] Sussillo D. Churchland M. M. Kaufman M. T. Shenoy K. V. (2015). A neural network that finds a naturalistic solution for the production of muscle activity. Nat. Neurosci. 18, 1025–1033. 10.1038/nn.4042 26075643 PMC5113297

[B46] Teulings H.-L. Contreras-Vidal J. L. Stelmach G. E. Adler C. H. (1997). Parkinsonism reduces coordination of fingers, wrist, and arm in fine motor control. Exp. Neurol. 146, 159–170. 10.1006/exnr.1997.6507 9225749

[B47] Thelen D. G. Anderson F. C. Delp S. L. (2003). Generating dynamic simulations of movement using computed muscle control. J. Biomech. 36, 321–328. 10.1016/S0021-9290(02)00432-3 12594980

[B48] Wolpert D. M. Diedrichsen J. Flanagan J. R. (2011). Principles of sensorimotor learning. Nat. Rev. Neurosci. 12, 739–751. 10.1038/nrn3112 22033537

[B49] Zajac F. E. (1989). Muscle and tendon: properties, models, scaling, and application to biomechanics and motor control. Crit. Rev. Biomed. Eng. 17:359. 2676342

