# Supplementary material for: Cortical Spiking Network Interfaced with Virtual Musculoskeletal Arm and Robotic Arm
Source: Front Neurorobot. 2015 Nov 25;9:13. doi: 10.3389/fnbot.2015.00013 (PMC4658435; doi:10.3389/fnbot.2015.00013)
Supplement: Supplementary file 2 [file Presentation1.PDF]

Supplementary Material: Cortical spiking network interfaced with virtual musculoskeletal arm and robotic arm

Salvador Dura-Bernal*, Xianlian Zhou, Samuel A Neymotin, Andrzej Przekwas, Joseph T. Francis and William W. Lytton

*Correspondence:
Salvador Dura-Bernal:
salvadordura@gmail.com

1 SUPPLEMENTARY METHODS

1.1 Single cell model equations and parameters

To model individual neurons we used event-driven, rule-based units which were able to reproduce key features found in real neurons, including adaptation, bursting, depolarization blockade, and voltage-sensitive NMDA conductance. Model neurons were characterized by a membrane voltage state variable (V_m), with a baseline value determined by a resting membrane potential parameter (V_{RMP}). This membrane voltage was updated based on one of three events: synaptic input, threshold spike generation, and refractory period. Synaptic inputs were modeled using reversal potentials, time constants and delays specific to each synapse type: AMPA, NMDA and $\mathrm{GABA_A}$. The equations for simulating synaptic inputs and action potentials are described below, and all parameter values can be found in Table 1. In addition to spikes generated by cells in the model, subthreshold Poisson-distributed spike inputs to synapses were used to provide ongoing activity, representing inputs from other neurons not explicitly simulated. The afferent weights and average firing rates used to stimulate the synapses of the different cell types is shown in Table 2.

Synaptic input was modeled by $V_n(t) = V_n(t_0) + w_s(1 - V_n(t_0)/E_i)e^{-\frac{t-t_0}{\tau_i}}$, where V_n is the membrane voltage of neuron n; t_0 is the synaptic event time (i.e., $t - t_0$ is the time since the event); w_s is the weight of synaptic connection s; E_i is the reversal potential of ion channel i, relative to resting membrane potential (where i = AMPA, NMDA, or $\mathrm{GABA_A}$;and τ_i is the receptor time constant for ion channel i).

A neuron fires an action potential at time t if $B_n > V_n(t) > T_n(t)$, where V_n, B_n, and T_n are the membrane voltage, blockade voltage, and threshold voltage, respectively, for neuron n. Action potentials arrive at target neurons at time $t_2 = t_1 + \tau_s v$, where t_1 is the time the first neuron fired, and τ_s is the delay due to synaptic conduction effects. After firing, a neuron cannot fire during the absolute refractory period, τ_A. Firing is reduced during the relative refractory period by two effects: first, an increase in threshold potential, $T_n(t) = T_n(t_0) + Re^{-\frac{t-t_0}{\tau_R}}$, where R is the relative refractory change in threshold voltage and τ_R is its time constant; and second, by hyperpolarization, $V_n(t) = V_n(t_0) - He^{-\frac{t-t_0}{\tau_H}}$, where H is the amount of hyperpolarization, and τ_H is its time constant.

Supplementary Table 1. Single cell model parameters for the different cell types and receptor types.

**Parameter**	**Value**
V_{RMP}	-63 or -65 depending on cell type
E_{AMPA}	65 mv
E_{NMDA}	90 mv
$E_{\mathrm{GABA_A}}$	-15 mV
τ_{AMPA}	20 ms
τ_{NMDA}	30 ms
$\tau_{\mathrm{GABA_A}}$ (somatic)	10 ms
$\tau_{\mathrm{GABA_A}}$ (dendritic)	20 ms
τ_s (somatic)	$U(1.8, 2.2)$ ms
τ_s (dendritic)	$U(3, 5)$ ms
B_n	-10 or -25 mV depending on cell type
T_n	-40 or -47 mV depending on cell type
τ_A (interneurons)	2.5 ms
τ_A (pyramidal)	5 ms
R (interneurons)	0.25
R (pyramidal)	0.75
τ_R (interneurons)	1.5 ms
τ_R (pyramidal)	8 ms
H (interneurons)	0.5 mV
H (pyramidal)	1 mV
τ_H (interneurons)	50 ms
τ_H (pyramidal)	400 ms

1.2 Network parameters

The probability of connection and starting weights between the different model populations is shown in Table 3.

Supplementary Table 2. Noise stimulation to synapses of the different cell types. Weight (W) values are afferent weights. Rate values are average stimulation frequencies in Hz (inputs are Poisson distributed). The asterisk beside the EM population indicates that the noise stimulation frequency depended on the phase, and was either $trainNoise$ Hz or $testNoise$ Hz.

Cell type	Synapse	W	Rate
ES	$GABA_A^{soma}$	1.875	100
ES	$AMPA^{dend}$	3.750	200*
ES	$GABA_A^{dend}$	1.875	100
IS	$GABA_A^{soma}$	1.875	100
IS	$AMPA^{dend}$	4.125	200
IS	$GABA_A^{dend}$	1.875	100
ILS	$GABA_A^{soma}$	1.875	100
ILS	$AMPA^{dend}$	3.000	200
ILS	$GABA_A^{dend}$	1.875	100
EM	$GABA_A^{soma}$	1.875	100
EM	$AMPA^{dend}$	3.938	200*
EM	$GABA_A^{dend}$	1.875	100
IM	$GABA_A^{soma}$	1.875	100
IM	$AMPA^{dend}$	4.125	200
IM	$GABA_A^{dend}$	1.875	100
ILM	$GABA_A^{soma}$	1.875	100
ILM	$AMPA^{dend}$	3.000	200
ILM	$GABA_A^{dend}$	1.875	100

1.3 Learning rule equations

The Critic's signal was calculated every $RLdt$ ms, based on the difference between the hand's and the target's location: the system was rewarded if the hand was getting closer to ther target, and punished if it was getting farther. We implemented a dopamine-based reward-modulated spike-timing dependent rule, and used eligibility traces to solve the credit-assignment problem. Synaptic tagging occurred where a postsynaptic spike followed a presynaptic spike within a time window of $t_{maxplast} = maxSTDPwin$ ms (spike-timing dependent rule). If a global modulatory signal was received within the time window of the eligibility trace, the trace was imprinted on the synapse, leading to an increase/long-term potentiation (for

Supplementary Table 3. Area (Pre: Presynaptic type; $Post$: Postsynaptic type) interconnection probabilities ($Prob$), and starting weights (W). * next to W represents plastic connection modified during learning. $Prob$ is the probability of a connection being included among all possible connections between the 2 areas. E cells used AMPA and NMDA synapses (NMDA, not displayed, had weights set at 10% of the colocalized AMPA synapse)

Pre	Post	Prob	W
P	ES	0.11250	4.000
ES	ES	0.05625	* 1.980
ES	IS	0.48375	* 1.150
ES	ISL	0.57375	* 0.575
ES	EM	0.33750	* 2.640
IS	ES	0.49500	4.500
IS	IS	0.69750	4.500
IS	ISL	0.38250	4.500
ISL	ES	0.39375	2.250
ISL	IS	0.59625	2.250
ISL	ISL	0.10125	4.5000
EM	ES	0.01125	* 0.720
EM	EM	0.05625	* 1.782
EM	IM	0.48375	*1.15
EM	IML	0.57375	* 0.575
IM	EM	0.49500	9.0000
IM	IM	0.69750	4.5000
IM	IML	0.38250	4.5000
IML	EM	0.39375	2.4900
IML	IM	0.59625	2.2500
IML	IML	0.10125	4.5000

reward), or decrease/long-term depression (for punishment) of the weight. The eligibility trace duration was $t_{maxelig} = maxEligTr$ ms, but, given the delay between motor commands and the virtual arm update was 50 ms, synapses tagged within $t_{minelig} = minEligTr$ ms of the global modulatory signal delivery were discarded, in order to avoid assigning credit/blame to spikes were not responsible for the action.

Weights $w(t)$ were updated utilizing weight scale factors, w_s:

$$
\begin{aligned}
w(t) &= w_0 \cdot w_s(t) \\
w_s(t+1) &= w_s(t) + \Delta w_s \\
\Delta w_s &= \begin{cases} w_{inc} \cdot (1 - w_s(t)/w_s^{max}) & \texttt{LTP} \\ -w_{inc} \cdot w_s(t)/w_s^{max} & \texttt{LTD} \end{cases}
\end{aligned}
$$

where w_s^{max} is maximum weight scale factor, and was set to $maxWscaling \cdot EEmaxW$ and $maxWscaling \cdot EImaxW$ times the synaptic weight of E E and E I baseline weights respectively; $w_0 = 1.0$ is the initial synaptic weight, and $w_{inc} = learnRate$ is the weight scale increment.

1.4 Parameters optimized via evolutionary algorithm

The sensorimotor system model includes a large number of parameters, many of which can be varied within an acceptable realistic range in order to obtain the desired results. We employed an evolutionary algorithm to optimize the value of 15 metaparameters, by using a fitness function that minimized the distance to target of the musculoskeletal arm trajectory. More specifically, we used a population size of 100 individuals (or candidate solutions) and applied the standard evolutionary strategy with $\tau = 1/\sqrt{2\sqrt{n}}$ and $\tau' = 1/\sqrt{2 \cdot n}$, where n is the number of parameters. Table 4 shows the list of parameters optimized, including the range of values explored and the value obtained for the top solution.

Supplementary Table 4. List of metaparameters optimized using evolutionary algorithm. For each metaparameter we include the range of values allowed and the value obtained by the top solution

Label	Description	Min allowed	Max allowed	Top solution
learnRate	RL learning rate	0.005	0.040	0.040
maxWscaling	Max weight scaling factor	0.7	1.5	0.7
EEmaxW	Max weight EE	5	10	7.85
EImaxW	Max weight EI	1.5	5	5.0
maxSTDPwin	STDP window duration (ms)	50	100	70
maxEligTr	Min eligibility trace window duration (ms)	50	150	62
minEligTr	Max eligibility trace window duration (ms)	25	75	30
EMmaxRate	Motor command threshold (spikes)	40	120	118
EMrateWin	Motor command window duration (ms)	30	120	76
RLdt	RL interval (ms)	50	110	55
maxExpMovdt	Max individual exploratory movement duration (ms)	100	1000	624
trainTime	Training phase duration (s)	90	480	360
trainNoise	Training phase background noise (Hz)	150	300	153
testNoise	Testing phase background noise (Hz)	50	150	99
